# Implementation, barriers and challenges of smoke-free policies in hospitals in Egypt

**DOI:** 10.1186/1756-0500-5-568

**Published:** 2012-10-15

**Authors:** Ghada Nasr Radwan, Christopher A Loffredo, Rasha Aziz, Nagah Abdel-Aziz, Nargis Labib

**Affiliations:** 1 Department of Public Health, Cairo University, Cairo, Egypt; 2 Lombardi Cancer Center, Georgetown University, Washington DC, USA

**Keywords:** Health care staff, FCTC, Smoke-free policies

## Abstract

**Background:**

Tobacco use is a serious public health challenge in North Africa, and health professionals play a vital role in tobacco control. In Egypt, limited data are available on the knowledge and attitudes of health care providers regarding tobacco control policies. Such data are especially relevant due to Egypt’s tobacco control laws, adopted in 2007, prohibiting smoking in hospitals and other public places. This study surveyed 49 senior administrative staff, 267 physicians, 254 nurses, and 109 administrative employees working in El-Kasr El-Aini Hospital in Cairo, assessing their knowledge and attitudes regarding Egypt’s tobacco control laws and barriers to their effective implementation in health care facilities. We also investigated the hospital’s compliance with smoke-free policies.

**Results:**

The majority (>90%) of the hospital workers knew that exposure to second-hand smoke is harmful to health. Physicians and nurses had a more favorable attitude towards the smoking ban when compared to administrative employees. Hospital staff identified the following barriers to successfully implementing the smoking ban: lax enforcement of tobacco control laws, the lack of penalties for violators, the lack of cessation programs, and the prevalence of smoking among physicians.

**Conclusions:**

Overall, smoke-free policies were poorly enforced in this large teaching hospital in Cairo, Egypt. Interventions to address the identified barriers to their implementation could include the provision of cessation training and services as well as effective communication programs to educate health care workers at all levels regarding the dangers of second-hand smoke exposure and effective measures for protection.

## Background

Tobacco use is a leading cause of preventable deaths throughout the world. Unless urgent action is taken, tobacco could kill one billion people in this century [1]. Prevalence rates of tobacco use are high throughout North Africa, and in Egypt around 38% of men use some form of tobacco product [2]. Of this percentage, nearly 32% smoke cigarettes, about 6% smoke shisha and almost 5% use smokeless (chewed) tobacco (ibid). In addition to cigarette consumption, Egypt is experiencing an upsurge in the prevalence of waterpipe (shisha) smoking, particularity among youth and women [3].

The WHO adopted the Framework Convention on Tobacco Control (FCTC) in 2003 to protect populations worldwide from the devastating health, economic, social and environmental hazards of exposure to tobacco and tobacco smoke [4]. In 2008, the WHO established MPOWER, a package of policies for tobacco control, which included raising taxes and prices, banning advertising, protecting people from second-hand smoke (SHS), and offering help to people who want to quit. These policies aim to reduce tobacco use and to help countries fulfill the promise of the FCTC. Egypt was an early signatory to the FCTC, having ratified it in February of 2005. In 2007, the adoption of new tobacco control laws which comprehensively banned smoking in all public places including health care facilities have added momentum to national tobacco control efforts and assisted Egypt in meeting its obligations under the FCTC. However a key challenge to the implementation of these laws is that they are inadequately enforced.

Health care facilities are among the most influential settings for modeling abstinence from smoking and promoting smoke-free environments. As such, health professionals have a prominent role to play in tobacco control. Their voices are heard throughout the social, economic and political arenas [5]. Hospitals can and should promote, implement and comply with tobacco control policies, particularly smoke free policies. However, this does not appear to be the case in Egypt, where the law on smoke-free workplaces enacted after the FCTC has yet to be enforced broadly across the society and within the health care sector specifically. A recent study conducted by Khan et al. 2012 revealed a high rate of smoking among medical students in Cairo University (46.7% were ever users of some form of tobacco). The research also revealed that there is no official policy banning smoking in the school buildings and clinics as reported by nearly half of the students; exposing the students, staff, patients and visitors to the detrimental health hazards of exposure to SHS [6]. Smoking bans, which include health care facilities, have also been implemented in Tunisia and Morocco, and countries throughout North Africa will face challenges similar to those in Egypt as they move forward with enforcing smoking bans [7].

In the current study, we aimed to assess the current knowledge of health care professionals and employees working in El-Kasr El-Aini Teaching Hospital regarding tobacco control policies in Egypt and the FCTC. We also aimed to assess their attitudes towards smoke-free polices, exposure to second-hand smoke (SHS) in the hospital as well as the degree to which smoke-free policies have been implemented in the hospital premises. In addition, we explored the perceived barriers and recommendations to successfully enforcing smoke-free policies in hospital settings. El-Kasr El-Aini Teaching Hospital located in Cairo is affiliated with the Faculty of Medicine and comprises more than 5200 beds, representing the largest medical institution in Egypt and indeed the Middle East. It is considered a symbol of the medical profession in Egypt and a stronghold of science and culture. Thus, the results of this study help to identify gaps in enforcing smoke-free policies in health care settings and add momentum to current tobacco control efforts by informing policy makers regarding existing challenges and recommended solutions.

## Methods

To assess the degree of implementation of smoke-free policies inÂ El-Kasr El-Aini Teaching Hospital a total of 149 observational checklists were completed by inspecting entrances, reception, waiting areas, patient wards, outpatient clinics, corridors, elevators, stairs, and physicians’, nurses’ and employees' rooms. The inspection was conducted by ten trained field workers using a standardized checklist to identify the presence of anti-smoking signs, posters, and cigarette butts.

A census of all hospital staff (senior staff, physicians, nurses and employees) was carried out and convenience samples were drawn from each staff category. The selection of subjects was based on their representation among the hospital’s staff. The estimated numbers of hospital staff were approximately 150 senior staff, 6000 physicians and 10000 nurses and other employees. We defined senior hospital staff as the head of the department and/or a senior professor from key hospital departments e.g. Chest, Cardiology, Internal Medicine. The overall inclusion criteria included being a permanent hospital staff member in the 4 categories listed above, availability during the time of the survey, and willingness to participate in the current study. Since we were unable to locate previous surveys assessing the level of compliance with smoke-free policies in workplaces and public places in Egypt, we estimated a sample size of approximately 400 subjects based on prevalence of workplace exposure to SHS reported in GATS of 2009 [2]. Over sampling was introduced to ensure adequate power to account for stratification of subjects into 4 groups and possible non response by busy hospital staff. We successfully enrolled a total of 679 participants representing 50% of the senior hospital staff (70% response rate) and 5% of physicians, nurses and employees (response rate = 82% for physicians, 89% for nurses and 92% for employees). A total of 49 senior administrative staff, 267 physicians, 254 nurses and 109 administrative employees were enrolled in the current study from March to May of 2007.

A 40-item face to face interview questionnaire was developed for the current study, which covered demographic and smoking behaviour, knowledge about the national tobacco control legislation, the FCTC and key tobacco control policies, attitudes towards implementing smoke-free policies in hospital settings (11 attitude questions), the degree of implementation of smoke-free policies at the hospital, perceived barriers, and suggested recommendations to implement smoke-free hospital policies. It was based on an instrument developed by the Institute for Global Tobacco Control of Johns Hopkins’ Bloomberg School of Public Health; the instrument was entitled ‘Implementing Hospital Smoking Policies: Background questions for hospital staff and employees’ (personal communication with Frances Stillman, 2003).

The responses to attitude questions presented three different options (agree, don’t agree and can’t decide). Responses to questions that assessed perceived barriers to and recommendations for the implementation of smoke-free policies included: very important, a little bit important and not important at all. Smoking status was assessed using the question, “Which statement describes you?” Response options were: I have never smoked a cigarette, I presently smoked cigarettes, I quit smoking cigarettes less than one year ago, and I quit smoking cigarettes more than one year ago. The estimated time to complete the questionnaire was 15–20 minutes. To accommodate for the busy schedules of senior staff, we developed a short version (23 items) of the questionnaire for interviewing senior staff. The data collection tools were developed in English, translated in Arabic, and then back-translated into English to ensure validity. They were also pilot tested before the actual data collection phase and some questions were modified accordingly. Official permission was obtained from the director of the hospital to conduct the current research and oral consent was obtained from the study participants.

Data analysis was carried out using the SPSS 12 software package. Comparisons of means across the groups of hospital staff were carried out using the ANOVA test. The chi-square test was used in analyses that entailed comparisons of proportions. Knowledge about key tobacco control policies and interventions was compared among physicians, nurses and employees and was assessed in relation to smoking status (never vs. current vs. ex-smokers). Attitudes towards the implementation of smoke-free policies (agree vs. disagree) were assessed among the hospital staff (physicians, nurses and employees) using a logistic regression analysis controlling for potential confounding factors such as smoking status (never vs. current vs. ex-smokers), age and gender (males vs. females). Odds ratios and 95% confidence intervals were calculated, and a p value of less than 0.05 was considered to be statistically significant. The project received ethical approval from the Research Ethics Committee of the Faculty of Medicine of Cairo University. Participants in the study were informed that participation was voluntary and that data would be treated as strictly confidential.

## Results

### Observational compliance with smoke-free policies

Nearly half of the inspected places in the study (entrances, reception, waiting areas, patient wards outpatient clinics, corridors, elevators, stairs, physicians’ nurses’ and employees rooms) had at least one anti-smoking sign (Table 1). The places where such signs were most likely to be absent (>70% of the time) were the patient wards, physician’s offices, nurse’s offices, and elevators. Educational and informational posters about smoking were virtually nonexistent, cigarette butts were prevalent at the entrances and waiting areas, and nearly one out of ten inspected places had ashtrays available. There was no designated smoking area in the hospital.

**Table 1:** **Distribution of anti-smoking signs, educational posters and cigarette butts in the hospital**

**Item inspected**	**No**		**Yes**		**Total**
	**N**	***%***	**N**	***%***	
***Presence of anti-smoking signs***	70	*47*	79	*53*	149
Entrance	7	*10.3*	61	*89.7*	68
Reception	24	*34.8*	45	*65.2*	69
Waiting areas	15	*21.4*	55	*78.6*	70
Patients’ wards	40	*74.1*	14	*25.9*	54
Employees' rooms	40	*58.8*	28	*41.2*	68
Physicians' rooms	43	*76.8*	13	*23.2*	56
Nurses' room	41	*78.8*	11	*21.2*	52
Outpatient clinics	27	*46.6*	31	*53.4*	58
Elevator	39	*73.6*	14	*26.4*	53
Corridors	22	*32.8*	45	*67.2*	67
Stairs	35	*59.3*	24	*40.7*	59
***Poster for anti-smoking laws***	147	*99.3*	1	*0.7*	148
***Educational posters***	143	*96.6*	5	*3.4*	148
***Presence of ashtrays***	129	*87.2*	19	*12.8*	148
***Presence of designated smoking areas***	149	*100*	0	*0*	149
***Presence of cigarette butts***					
Entrance	87	*59.6*	59	*40.4*	146
Reception	111	*78.7*	30	*21.3*	141
Waiting areas	97	*67.4*	47	*32.6*	144
Patient wards	130	*92.2*	11	*7.8*	141
Employees' rooms	114	*80.9*	27	*19.1*	141
Physicians' rooms	116	*82.3*	25	*17.7*	141
Nurses' room	129	*91.5*	12	*8.5*	141
Outpatient clinics	104	*75.4*	34	*24.6*	138
Elevator	113	*82.5*	24	*17.5*	137
Corridors	98	*68.5*	45	*31.5*	143
Stairs	105	*75*	35	*25*	140

Furthermore, we explored the existence and enforcement of smoke-free policies within the hospital premises by interviewing senior and other hospital staff. 78% of senior staff reported the presence of these policies, but less than half (48%) reported that these policies were enforced within the hospital premises. Nearly 70% claimed that selling tobacco products was prohibited within the hospital. Only 2% mentioned that there were cessation programs to aid physicians and employees in quitting smoking. Similar findings resulted from interviews with other hospital staff groups, where a large proportion reported the existence (61.6%) of smoke-free policies, however enforcement was reported by only one third of the staff (35.5%). In addition, the interviewed hospital staff reported that visitors (87%), employees (86%), physicians (79%), patients (65%), and nurses (54%) smoked within the hospital premises. 59.5% claimed that selling tobacco products was prohibited within the hospital premises. Only 13% mentioned that there were cessation programs to aid physicians and employees in quitting smoking. Nearly half of the interviewed subjects (53%) reported the presence of anti-smoking signs on the hospital premises (data not shown).

### General characteristics, smoking habits of hospital staff and their knowledge of tobacco control policies and FCTC

A total of 49 senior administrative staff, 267 physicians, 254 nurses and 109 administrative employees were enrolled in the current study. The majority of the senior staff interviewed were males (71.4%), and their mean age was 51.1 + 6.8 years (ranged 34–60 years). One out of five male senior staff was a current cigarette smoker (20%). None of the interviewed female senior staff were identified as smokers (mean age was 51.1 + 6.8 years; ranged from 34–60 years). Among other groups of hospital workers (physicians, nurses and employees), there were 254 (41.8%) males. The mean age was 29.7 ± 8.8 years. When the smoking status was stratified by gender , the prevalence of current smoking among male physicians, nurses and employees was 12.5%, 30.0% and 28.1%, respectively (p < 0.05). The overall prevalence among females was less than 1%. The distribution of the study group by occupation, age, sex and smoking status is shown in Table 2.

**Table 2:** **The distribution of the study group by occupation, age, sex and smoking status**

**Item**		**Physicians**		**Nurses**		**Administrative staff**		**P-value**
Age (N=608)	Mean ( SD)	29.6	(7.4)	27.9	(8.6)	34.3	(10.7)	.000**
Sex (N=608)	Males	160	61.8%	30	12.2%	64	62.1%	.000*
Smoking status among males (N=254)	Never	132	82.5%	18	60.0%	41	64.1%	0.02 *
	Current	20	12.5%	9	30.0%	18	28.1%	
	Ex smoker	8	5.0%	3	10.0%	5	7.8%	

*Chi-Square test.

** ANOVA test.

The vast majority of staff (>90%) were knowledgeable about the laws which ban smoking in public places. A fairly large percentage (>70%) were aware of laws prohibiting the sale of tobacco products to minors less than 18 years, the Fatwa (religious ruling) that prohibits smoking and the laws banning all forms of tobacco advertising, promotion and sponsorship and requiring health warnings covering at least 30% of the main display area of the cigarette pack. More than one third of the staff knew that there was a law that bans smoking on public transport, and 3-6% of staff knew about the FCTC (data are not shown).

### Knowledge of hospital staff on impact of exposure to SHS and key tobacco control policies and their attitudes towards smoke-free policy implementation on the hospital premises

We assessed the participants’ knowledge about the impact of SHS exposure in the workplace as well as some key tobacco control policies. 90% of the study group correctly affirmed the question “exposure to SHS in the workplace is a significant cause of tobacco related diseases”. However, 62% reported that separate ventilation can offer the same protection as a smoking ban and can be a good alternative to bans. The gaps in knowledge were identified in the areas related to the effectiveness of health warnings on the cigarette packages (10.5%), awareness measures of tobacco risks (15%), and youth access provisions (39%). There was no significant difference in the knowledge between physicians, nurses and employees in most of the knowledge items examined. Physicians were least knowledgeable about health warning effectiveness, and nurses had the least knowledge about effectiveness of tobacco taxation (Table 3). Moreover, when the same knowledge items were examined in relation to smoking status, a significantly higher level of knowledge was only found in the question inquiring about whether exposure to tobacco smoke in the workplace is a significant cause of tobacco related-diseases. In response to this question, a significantly higher proportion of never and ex-smokers reported that exposure to tobacco smoke in the workplace is a significant cause of tobacco related-diseases (Table 3).

**Table 3:** **Knowledge about key tobacco control policies by occupation and smoking status**

**Item**	**Correct answer**	**Physicians N*%***	**Nurses N*%***	**Employees N*%***	**Total N*%***	P-value*	**Never smoker N*%***	**Current smoker N*%***	**Ex-smoker N*%***	**Total N*%***	P-value*
Exposure to tobacco smoke in the workplace is not a significant cause of tobacco-related diseases (N= 625 )	False	246	227	98	571	.689	514	40	17	571	.000
		***92.5%***	***90.4%***	***90.7%***	***91.4%***		***92.4%***	***76.9%***	***100 %***	***91.4%***	
Designated smoking areas in the same room are effective in protecting non-smokers and workers from the hazards of tobacco smoke (N=624 )	False	188	199	82	469	.072	419	36	14	469	.590
		***70.7%***	***79.3%***	***76.6%***	***75.2%***		***75.4%***	***70.6%***	***82.4%***	***75.2%***	
Separate rooms with separate ventilation offer almost the same amount of protection as smoking bans and are a good alternative to bans (N= 626 )	False	152	161	73	386	.082	352	26	8	386	.079
		***56.9%***	***63.9%***	***68.2%***	***61.7%***		***63.2%***	***50.0%***	***47.1%***	***61.7%***	
Tobacco product advertising has no effect on consumption (N=621)	False	183	149	65	397	.084	360	26	11	397	.187
		***68.8%***	***59.6%***	***61.9%***	***63.9%***		***65.0%***	***52.0%***	***64.7%***	***63.9%***	
Because of all the publicity about how harmful tobacco use is, anyone who starts using tobacco these days is fully aware of the risks (N= 623 )	False	39	36	19	94	.696	80	12	2	94	.204
		***14.7%***	***14.4%***	***17.8%***	***15.1%***		***14.4%***	***23.5%***	***11.8%***	***15.1%***	
Warnings/messages on tobacco product packages, while attention getting, are not effective in motivating users to quit (N=621)	False	18	31	16	65	.028	57	7	1	65	.614
		***6.8%***	***12.4%***	***15.0%***	***10.5%***		***10.3%***	***13.7%***	***5.9%***	***10.5%***	
Tax increases will result in smuggling and nothing can be done about it (N=623)	False	128	135	44	307	.092	275	26	6	307	.504
		***48.5%***	***53.6%***	***41.1%***	***49.3%***		***49.6%***	***50.0%***	***35.3%***	***49.3%***	
Youth access provisions tend to be difficult to enforce (N=617)	False	114	92	38	244	.276	216	24	4	244	.219
		***43.2%***	***37.1%***	***36.2%***	***39.5%***		***39.3%***	***47.1%***	***23.5%***	***39.5%***	
The most cost effective control option in all regions is taxation on tobacco products (N=626)	True	146	110	60	316	.019	281	24	11	316	.445
		***54.7%***	***43.7%***	***56.1%***	***50.5%***		***50.4%***	***47.1%***	***64.7%***	***50.5%***	

*Chi-Square test.

An agreement with the statement “hospitals should be smoke-free” was professed by all interviewed senior staff, and more than 90% of them believed that a smoke-free hospital environment would improve the quality of care that patients receive (data not shown). Since several research studies have demonstrated that smokers are less supportive of smoking bans than non smokers, we explored whether our findings are consistent with such findings [8]. We found that current smokers had generally less favourable attitudes toward some aspects of smoke-free polices, their impact and implementation. For example, a higher proportion of current smokers believed that hospital employees who work in offices or areas removed from direct patient care should be allowed to smoke (29% vs. 15%, p > 0.05). Furthermore, agreement that the smoking habits of health professionals influence others was significantly lower in current smokers than in non-smokers (71% current vs. 100% non-smokers, p value <0.05) (data are not shown).

The attitudes of other staff members towards the implementation of smoke-free policies were assessed using an inventory of 11 questions, which compared physicians, nurses and administrative employees, adjusting for age, gender and smoking status. A significantly higher proportion of physicians thought that exposure to SHS was unhealthy to nonsmokers (OR 4.6, CI 1.4-15). Generally, more favorable attitudes towards the implementation of smoke-free hospital policies were professed among health professionals (physicians and nurses) compared to administrative employees. Physicians were more likely to disagree with the statements “smoke-free policies are hard to enforce,” “smoke-free policies are unfair to smokers,” and “hospitals with smoke-free policies are likely to lose patients”. Physicians were more likely to support the need for cessation programs to be offered to employees (OR 6.7, CI 1.6-27.3, p < 0.01). A significantly higher proportion of health professionals (physicians and nurses) compared to employees expressed that a smoke-free environment would positively influence their job performance and the public image of their hospital (Table 4).

**Table 4:** **Attitudes of hospital staff towards smoke-free policies and second-hand smoke exposure**

**Item**		**Number**	**Percent**	Adjusted OR*	**95% CI**	
A smoke-free hospital would improve the quality of care the patient receives (Agree)	Admin**	90	82.6%	1.0		
	Physicians	223	83.8%	1.4	0.63	3.1
	Nurses	227	90.4%	2.2	0.84	5.7
Smoke from someone else's cigarette is unhealthy for non-smokers (Agree)	Admin	97	89.8%	1.0		
	Physicians	260	97.7%	4.6	1.41	15.1
	Nurses	245	97.2%	3.6	1.04	12.6
The smoking habits of health professionals influence others (Agree)	Admin	100	91.7%	1.0		
	Physicians	258	97.0%	3.0	0.93	10.0
	Nurses	236	93.7%	1.7	0.51	5.9
Cessation programs should be offered to employees (Agree)	Admin	101	92.7%	1.0		
	Physicians	254	95.5%	6.8	1.68	27.3
	Nurses	239	95.2%	2.2	0.60	7.7
A hospital should be a smoke-free environment (Agree)	Admin	105	97.2%	1.0		
	Physicians	256	96.2%	1.2	0.30	4.6
	Nurses	246	97.6%	1.4	0.27	7.4
Hospital employees who work in offices or areas removed from direct patient care should be allowed to smoke (Disagree)	Admin	76	69.7%	1.0		
	Physicians	196	73.7%	1.4	0.77	2.6
	Nurses	155	61.3%	0.7	0.36	1.3
A smoke-free policy is hard to enforce (Disagree)	Admin	46	42.2%	1.0		
	Physicians	142	53.6%	2.1	1.28	3.5
	Nurses	104	41.6%	1.2	0.66	2.0
Having a smoke-free policy is unfair to smokers (Disagree)	Admin	90	84.1%	1.0		
	Physicians	234	88.0%	2.6	1.08	6.1
	Nurses	223	88.8%	1.2	0.47	3.1
Hospitals with smoke-free policies are likely to lose patients (Disagree)	Admin	86	79.6%	1.0		
	Physicians	205	77.1%	2.6	1.24	5.4
	Nurses	186	73.5%	0.9	0.45	2.0
Smoking bans at hospitals would positively influence job performance (Agree)	Admin	59	54.6%	1.0		
	Physicians	190	71.7%	2.0	1.21	3.3
	Nurses	190	75.7%	2.1	1.20	3.6
Smoking bans at hospitals would positively affect the public image of the hospital (Agree)	Admin	74	68.5%	1.0		
	Physicians	245	92.8%	5.5	2.79	10.8
	Nurses	226	89.7%	3.3	1.63	6.6

*Logistic regression **Administrative employees.

Smoking cessation interventions offered in the hospital were also assessed in the current study. Our results revealed that some cessation services were more likely to be provided compared to others. For example, a high proportion of physicians stated that they ask about their patients’ smoking status (70%) and advise them to quit (74%). However, services such as encouraging a quit date (44%), arranging for follow up visits (28%) and providing quit medications (39%) were mentioned by less than half of the interviewed physicians.

### Perceived barriers and recommendations to successfully enforcing smoke-free policies in hospital settings

In addition to the recommendations of senior staff to implement smoke-free hospital policies, they also expressed the urgent need to provide smoking cessation programs for physicians and employees (85.4%) and to provide smoking cessation training for physicians (84.8%). These were followed by the need to establish a tobacco control committee (81%) and to provide the necessary logistical support for the implementation of these policies and programs (75%).

Consistent with the findings obtained from surveying senior staff, the physicians, nurses and employees pointed out the utmost importance of providing smoking cessation programs to help those willing to quit smoking (79%), the provision of smoking cessation training for physicians (78%), the need to establish a tobacco control committee in the hospitals (78%) and the provision of logistical support for tobacco control programs and policies (77%). Provision of smoking cessation programs for physicians and employees was recommended by a higher proportion of nurses and physicians compared to administrative staff (80%, 79% vs. 78%, p < 0.05), while provision of educational programs for employees and staff was recommended by a higher proportion of administrative staff compared to nurses and physicians (77% vs. 59% and 60%, p < 0.05). Lax enforcement of smoke-free policies (93%), the lack of penalties for violations (96%), the absence of smoking cessation programs to help those willing to quit (92%) as well as the fact that physicians were smokers (96%) were reported by the study participants as the major obstacles to the implementation of smoke-free policies in hospital setting. The statement “Smoking is not a priority problem” was reported by more than half of administrative employees (53%) as one of the obstacles, compared to nearly one third of physicians and nurses (35% and 39%, p < 0.05) (data are not shown).

## Discussion

Egypt has the largest population of tobacco users in the Arab world. As with many developing countries in the early stages of the smoking epidemic, tobacco use in Egypt is largely concentrated among males, with nearly 40% of adult males smoking some form of tobacco in 2009 [2]. Female smoking prevalence is low, but has increased more rapidly than male prevalence in recent years as social norms against women’s smoking have weakened [9].

The role of hospitals and other medical institutions is crucial in promoting healthy behavior in the community. Health professionals themselves play a particularly important role in tobacco control. [10]. Health professional smoking prevalence varies widely around the world, reflecting sociodemographic patterns of tobacco use [11]. In the current study, the lowest smoking prevalence among the different study groups was witnessed in physicians (12.5% among male physicians, 20% among senior staff, 28% among nurses and 30% among employees) which is consistent with similar research elsewhere [12,13]. Comparing the prevalence of this study with the data obtained from the Global Health Professional Survey conducted in five countries in the Eastern Mediterranean Region between 2002 and 2004, including Egypt, smoking prevalence was around 32% among male respondents, which included physicians, dentists, nurses and others working in supporting professions [14].

In our study the prevalence of smoking was much higher among senior staff compared to physicians (20% vs. 12.5%). This was consistent with the findings of the international review conducted by Smith and Leggat, 2007, which found that many authors documented age-related differences in physicians' smoking rates, with older physicians for the most part more likely to be current smokers [15]. However in some countries such as China [16], Japan [17], Mexico [18] and India [19] tobacco use was actually more prevalent in younger physicians, which presents a major challenge for these countries. Surprisingly, we identified no significant difference in knowledge among physicians, nurses and administrative employees. These findings point to the utmost need to target health professionals as a group, through awareness and training programs. In addition, we observed that the only tobacco control policy statement on which knowledge differed among the 3 smoking status groups was the health hazards of exposure to SHS in the workplace, where a significantly lower proportion of current smokers agreed with the statement compared to the other two groups (ex and never smokers). This is consistent with the findings of Duaso et al. 2006, who compared the beliefs of smokers and non-smokers on the risks of SHS and found that perceived risks were significantly higher among non-smokers [20].

Our results revealed that attitudes towards the implementation of the smoke-free hospitals rule were more favorable among health professionals (physicians and nurses) compared to administrative employees. They were more likely to believe that smoke-free policies were feasible and could impact employees in the hospital. Survey data in many countries have demonstrated support for smoke-free policies and tobacco control. For example, health professionals responding to the Health Professionals Survey in 2005 overwhelmingly supported banning smoking in enclosed public places (97%), using large-print health warnings on cigarette packaging (87% of smokers and 93% of non-smokers), banning sales to minors (97%), banning sport sponsorship by the tobacco industry (92%), banning tobacco advertising completely (97%), and making hospitals completely smoke-free (96%) [21].

Local data on support for smoke-free policies are scarce in Egypt. In 2007, the Information and Decision Support Center (IDSC) launched a telephone survey to measure citizens’ attitudes towards cigarette smoking and knowledge about tobacco harm. The results revealed that the majority of respondents (94% and more) believed that hospitals, public transportation, restaurants, government departments, companies and universities, among other places, should ban smoking. In addition, 88% of respondents supported imposing a financial penalty on violators in public areas, and 83% said that providing free medicine to help smokers quit would also be an effective solution [22]. This was consistent with the growing evidence that smoke-free policies are popular and supported by the majority of the population (both smokers and nonsmokers) [23].

The vast majority of the study group believed that the smoking habits of health professionals influence others. Furthermore, the smoking habits of physicians were identified by all groups as one of the most important barriers to the implementation of smoke-free hospital policies. These findings are consistent with the findings of Hodgetts et al. 2004 [24]. They found that more than 80% of the interviewed physicians and nurses agreed with the statements, “Health professionals serve as role models for their patients and the public,” “Health professionals should set a good example by not smoking,” and “Patients’ chances of quitting smoking are increased if a health professional advises him or her to quit.” Similarly Czajkowska-Malinowska et al. 2008, reported that the majority of pulmonologists (81.1%) thought that physicians’ attitudes influence patients’ behavior and more than a half (55.7%) thought that smoking by physicians is not in accordance with physicians’ ethics [12].

Despite the claim by more than half of our studies’ participants that there was a smoke-free policy in the hospital, only one third reported that it was enforced. In addition, the vast majority of participants (>88%) identified “no enforcement of smoke-free policies” as a barrier to successful implementation of smoke-free hospital policies. Poor enforcement of smoke-free policies in hospital and workplace settings has been reported in many studies in developing countries. In China a survey of 3552 hospital-based physicians from six Chinese cities reported that only one third cited good implementation of smoke-free workplace policies, and a similar proportion of current physician smokers had smoked in front of their patients [13].

Cessation support and services are important measures to achieving a 100% smoke-free health care facility [25]. In the current study only half of the interviewed staff reported that they assisted patients who wanted to quit and 39% prescribed quit medications. Nevertheless, some of the most important barriers reported to effective enforcement of tobacco control hospital policies were the lack of cessation training for physicians and the absence of cessation services for smokers willing to quit. The poor provision of cessation support could be in part due to the absence of knowledge and skills among physicians on how to counsel smokers. This is supported by data gathered from the Health Professional Survey, which revealed that only 20% of medical students received formal training in smoking cessation counseling. Only half of respondents reported feeling “well prepared” to give counseling on smoking cessation, while another 30% reported feeling “somewhat prepared” [21]. Consistently, our results showed that provision of cessation services and cessation training for physicians were among the most important recommendations for effective implementation of smoke-free hospital policies. Health professionals surveyed by Hodgetts et al. 2004, reported similar findings. They emphasized the importance of providing health professionals with specific training on cessation techniques [24].

In the current study we identified many barriers to implementing effective tobacco control policies in hospitals. Smoking by physicians, lax enforcement, a lack of penalties for violators and lack of cessation treatment were among the barriers reported by most of the interviewed staff. In 2005 the WHO published a report on the role of health professionals in tobacco control which revealed many barriers to the participation of health professionals in tobacco control. Among these barriers were the lack of knowledge and skills about tobacco and tobacco control and lack of awareness of the epidemiological aspects of tobacco use and its impact on global health – these barriers hinder tobacco professionals from joining the battle against the tobacco epidemic [5].

The current study has several limitations related to its cross-sectional design including issues of recall and response bias and the reliance on self-reported data. In addition, the use of convenience sampling was a limitation of the study. However the rigorous design and the execution of the survey by public health trained field workers were some of the strengths of this study. An additional limitation is related to the focus on one teaching hospital; due to budget limitations; which might not reflect the status of the implementation of smoke-free policies in other health care settings (such as general or private hospitals). This warrants future research targeting such settings.

## Conclusion

In conclusion, the findings of this study prompt immediate action to enhance education regardingÂ tobacco controlÂ for health care workers in Egypt. This should begin with the integration of effective tobacco control policies and cessation support into the curricula of medical and nursing schools as well as provision of cessation counseling training for physicians and nurses. Health care worker development is essential to prepare physicians in order to assist millions of smokers in quitting, to shift professional and societal norms towards cessation, and to advocate for and support comprehensive tobacco control in Egypt.

## Competing interests

The authors declare that they have no competing interests.

## Authors’ contributions

GNN: co-investigator of the study, carried out study design and developed tools was the primary writer of the manuscript and carried out data analysis and presentation. CAL: consulted in all steps of the study and participated in manuscript writing. RA: supervised data management and data entry and participated in the field work. NAA: participated in tools development and field work. NA: principal investigator of the study, supervised all study procedures and data collection and obtained ethical and official approvals as well as funding for the study. All authors read and approved the final manuscript.
